# Dr. Frank P. Foster

**Published:** 1908-12

**Authors:** 


### Dr. Frank P. Foster.

IN the November issue of the JOURNAL, the newly created Medical Reserve Corps of the Army was dealt with in some detail. In that article reference was made to the fact that Dr. Frank P. Foster, among others, had been appointed a medical officer in that corps, with the rank of first lieutenant, the highest rank bestowed by an initial commission. Dr. Foster is the widely known editor of the *New York Medical Journal*, and through its columns has been a consistent advocate of reforms in the medical service of the army, as well as an ardent supporter of the policies of the surgeon general relating to placing the medical corps upon a high plain of preparedness, that it may always be in complete readiness for any sudden demand for its services.

One Saturday afternoon, in the late November days, a group of men known as the "Fensophs" gathered in the club rooms of the organisation and celebrated Dr. Foster's appointment in a most appropriate manner. The meeting was called ostensibly to celebrate Dr. Foster's birth day, he himself having been for a long time dean of the Fensophs. The real object of the meeting however, was to present to the new medical officer a sword appropriate to his rank and corps, this purpose being adroitly concealed from the recipient, as we have already hinted. The subterfuge worked admirably; the dean was taken completely by surprise in his own camp when Mr. Newell, one of the gifted Fensoph's arose and delivered a fitting presentation speech, at the close of which advancing to the head of the table and placing the sword, with its beautifully embossed blade, containing the names of the donors, in the hands of the dean, now a medical officer in the United States Army Reserve Corps.

The club rooms were appropriately decorated with army colors, flags, and other fitting insignia. The feast was made up of a grouping of good things to eat, drink and smoke; there was a camaraderie of fellowship in the air, the speeches were sparkling and bright, and altogether it was a day long to be remembered by the Fensophs in the honor they did their beloved Dean.

DECLARATION was made recently by Dr. William H. Maxwell, city superintendent of schools in New York, says the *Evening Mail*, that adenoids in children are so alarmingly baneful that they produce depravity in boys and girls who have them.

Dr. Maxwell made this statement in asserting that it was imperative that the Board of Education be clothed with power to compel parents of children so affected to have the adenoid growths removed. If they are permitted to remain the child is almost certain to develop criminal tendencies, said Dr. Maxwell. "We have found that pupils with adenoids and enlarged tonsils, are the ones who make the noise in the classroom, defy their teachers and are generally incorrigible.

OPERATION MAKES BAD BOY GOOD.

In those children who have had the adenoid growths removed there has been an immediate improvement in behavior and intelligence, while in children whose parents refuse to permit them to be treated, the child grows steadily worse in disposition and morals. So serious are the evils of this particular throat affliction that to them can be traced criminal tendencies which develop in boys after they have grown up.

This is a topic of sufficient importance to engage the attention of the school inspectors in Buffalo, and should be taken up at an early period in each child's school life.

---

"Moral Anesthesia" is the latest defence announced for crime involving plotting against life. This plea was offered recently in Chicago, for a girl charged with attempting to plot the murder of her mother, and was accepted by the court. A deadening of the moral perceptions would serve to cover a multitude of crimes, could it be established as an orthodox answer in the courts. It sounds better than the hackneyed "insanity"—insane while committing the crime, sane a moment before and a moment afterward, does not fit scientific medicine, howevermuch courts and juries may be pleased to accept it.

---

Not more than 25 per cent. of parents who are notified by the school board in New York, that their children require treatment of one kind or other for eyes, teeth or throat comply with the request to have the children looked after.

It is because of this fact, and the baneful effects of adenoid growths in the throat, that it is imperative that there should be legislation investing the school authorities with absolute power to compel children to have these growths removed.

The law gives the board the right to put away boys and girls who are ungovernable. They are sent from school to school to see if changed surroundings will benefit them, and when all else fails they are sent to reformatories. But before they are put away they are examined for the insidious growths, and in many cases their removal restores the child to reasonableness and good behavior and brings about increased intelligence and capacity for study.

Professor William E. Castle, of Harvard University, asserts that he has discovered a mathematical and precise rule which will determine the sex of higher animals. He has bred two freak varieties of guinea pigs, which he says give to the world the first tangible evidence that fixed laws govern sex production.

---

Dr. William Warren Potter of Buffalo, was elected president of the State Board of Medical Examiners and Dr. William S. Searle of Brooklyn, vice-president, at the annual meeting held at Albany, November 16, 1908.

The board placed itself on record in favor of the six year combined baccalaureate and medical course. Arrangements were made for the preparation of a medical syllabus in keeping with the suggestions of prominent medical educators, and the committee of which Dr. Lee H. Smith of Buffalo is chairman was continued in power. The assignments to the various subheads of medicine were voted as follows:

Physiology, Dr. R. H. Williams of Rochester; anatomy, Dr. William S. Ely of Rochester; hygiene and sanitation, Dr. Eugene Beach of Gloversville; chemistry, Dr. F. S. Farnsworth of Plattsburg; surgery, Dr. F. S. Crandall of New York; obstetrics and gynecology, Dr. W. W. Potter of Buffalo; bacteriology, Dr. F. W. Adriance of Elmira; pathology, Dr. Lee H. Smith of Buffalo; diagnosis, Dr. William S. Searle of Brooklyn.

The following were elected members of the question committee: Drs. Smith, Potter, Adriance and Williams.

---

In explanation of the wrecking of railway trains, some of the speakers at the recent annual meeting of the New York and New England Association of Railway Surgeons, said love, liquor, gambling, and improper signals were some of the things responsible for many fatal railroad accidents.

Dr. R. W. Corwin of Pueblo, Col., told of a young engineer, who, through jealousy, neglected his work, with the result that his train was wrecked. He told of another engineer who, while worrying over the fact that the night before he had gambled away his pay check, backed his train into an excursion train. The speaker denied that railroads overwork their men by deliberate choice and said it would be decidedly false economy for them to do so.

"Sometimes drink is mistaken for brain fag and overwork," said he. "The public has a right to demand from railway systems that employees be given sufficient rest, but that does not go far enough. The employees should take rest during the time allotted to them, but when they do not, what then? The employee who uses his recreation for gambling, drinking and smoking to excess is no more fit for work than is the man who has been on duty sixteen or twenty hours."

---

A SENTENCE to a term of imprisonment for "substitution" in a drug store may seem to some to be harsh, (*New York Tribune*), but it must be remembered that the offence is in any case swindling and is also potentially one of the gravest that can be committed. In the case now in point the culprit refilled with ordinary water bottles which had contained water from a special spring. Perhaps no harm was done to life or health. But the purchaser of the refilled bottles was cheated, for he did not get that which he ordered and for which he paid, and the proprietor of the original contents of the bottles was injured, for the sale of the spurious stuff militated against the good repute of the genuine. For such an offence a man deserves severe punishment. But it must be remembered that the practice of substitution, if carried further, as it has been in not a few cases, might involve such vitiation of important prescriptions as would in the gravest manner affect the health and even the life of a patient. For such an offence three months in jail is really a light punishment. If the disposition of the present case, which has been before the courts for years, shall cause a general abandonment of a practice which has been by far too common a great gain for the public welfare will be attained.

